# Supplementary material for: Risk of Adverse Pregnancy Outcomes for Women with IBD in an Expert IBD Antenatal Clinic
Source: J Clin Med. 2022 May 22;11(10):2919. doi: 10.3390/jcm11102919 (PMC9146846; doi:10.3390/jcm11102919)
Supplement: Supplementary file 1 [file jcm-11-02919-s001.zip › jcm-1720587-supplementary.pdf]

Supplemental Tables

Supplemental Table S1 – Biologic, ASA and Thiopurine Exposure and Neonatal Outcomes

Biologic exposure	N (%)	Total per outcome	Vs Other IBD Medication			Vs No Medication		
			P	RR	95% CI	P	RR	95% CI
**Preterm**	7 (18.4)	**15**	0.014	3.29	1.23-8.79	0.075	4.97	0.65-38.11
**LBW**	3 (7.89)	**11**	0.33	1.97	0.49-7.88	0.66	0.71	0.16-3.26
**VLBW**	1 (2.63)	**3**	0.37	3.29	0.21-51.34	0.81	0.71	0.046-10.87
**SGA**	3 (7.89)	**13**	0.64	1.35	0.39-4.72	0.79	1.27	0.23-7.03
**NICU**	1 (2.63)	**5**	0.89	1.17	0.13-10.88	0.42	-	-
**Stillbirth**	1 (2.63)	**4**	0.85	1.24	0.13-11.55	0.37	0.36	0.034-3.72
**TOTAL Biologic exposed patients**	**38 (100)**							
**ASA exposure**	**N (%)**	**Total per outcome**	**Vs Other IBD Medication**			**Vs No Medication**		
			**P**	**RR**	**95% CI**	**P**	**RR**	**95% CI**
**Preterm**	6 (9.23)	**15**	0.63	1.28	0.47-3.53	0.38	2.4	0.31-18.9
**LBW**	4 (6.15)	**11**	0.99	1.00	0.30-3.29	0.41	0.55	0.13-2.31
**VLBW**	0 (0)	**3**	0.28	-	-	0.12	-	-
**SGA**	7 (10.77)	**13**	0.10	2.44	0.81-7.36	0.53	1.60	0.36-7.21
**NICU**	3 (4.62)	**5**	0.10	5.24	0.56-49.21	0.27	-	-
**Stillbirth**	1 (1.54)	**4**	0.63	0.58	0.062-5.51	0.15	0.21	0.020-2.20
**TOTAL ASA exposed patients**	**65 (100)**							
**Thiopurine exposure**	**N (%)**	**Total per outcome**	**Vs Other IBD Medication**			**Vs No Medication**		
			**P**	**RR**	**95% CI**	**P**	**RR**	**95% CI**
**Preterm**	7 (11.67)	**15**	0.17	1.98	0.73-5.39	0.24	3.09	0.40-23.81
**LBW**	3 (5)	**11**	0.65	0.74	0.20-2.70	0.30	0.45	0.097-2.09
**VLBW**	1 (1.67)	**3**	0.99	0.99	0.092-10.72	0.81	0.71	0.046-10.87
**SGA**	3 (5)	**13**	0.47	0.63	0.18-2.24	0.71	0.72	0.13-4.08
**NICU**	3 (5)	**5**	0.073	6	0.64-56.32	0.25	-	-
**Stillbirth**	2 (3.33)	**4**	0.48	1.98	0.29-13.73	0.40	0.45	0.067-3.03
**TOTAL Thiopurine**	**60 (100)**							

exposed patients								

Supplemental Table S2 – Disease Activity and Neonatal Outcomes

Moderate/Severe disease activity during pregnancy	N (%)	Total per outcome	P	RR	95% CI
**Preterm**	4 (16.67)	**15**	0.12	2.35	0.81-6.78
**LBW**	2 (8.33)	**11**	0.63	1.44	0.33-6.25
**VLBW**	1 (4.17)	**3**	0.31	3.23	0.30-34.25
**SGA**	4 (16.67)	**13**	0.056	2.87	0.96-8.59
**NICU**	1 (4.17)	**5**	0.66	1.62	0.19-13.84
**Stillbirth**	1 (4.17)	**3**	0.31	3.23	0.30-34.25
**TOTAL Mod/Severe disease activity during pregnancy**	**24 (100)**				
